# Evolving strategies in pulmonary arteriovenous malformation embolization

**DOI:** 10.1186/s42155-026-00702-x

**Published:** 2026-05-14

**Authors:** Usama Anwar, Shermeen Ahmed, Kanishk Aggarwal, Maslahuddin Hayat Alhaque Roomi, Tuba Khan, Rachita Kour

**Affiliations:** 1 Mercy Catholic Medical Center, 1500 Lansdowne Ave, Darby, PA 19023 USA; 2 University of Maryland Medical Center Midtown Campus, 827 Linden Ave, Baltimore, 21201 USA

**Keywords:** Pulmonary arteriovenous malformation, Endovascular embolization, Vascular plugs, Microvascular plugs, Low-profile braided occlude, Coil embolization, Nidus embolization, Hereditary hemorrhagic telangiectasia, Dual-energy CT, Interventional radiology

## Abstract

Pulmonary arteriovenous malformations (PAVMs) cause right-to-left shunting with risks of hypoxemia and paradoxical embolization. Endovascular embolization is the standard treatment, but long-term durability depends on anatomy, flow characteristics, device selection, embolization strategy, and follow-up definitions. This narrative review synthesizes contemporary evidence on embolic devices—including coils, vascular plugs, microvascular plugs, low-profile braided occluders, and liquid embolics—along with adjunctive techniques such as balloon occlusion, nidus-directed embolization, and cone-beam CT guidance. Available retrospective data suggest that plug-based strategies may be associated with lower persistence in anatomically suitable lesions, whereas coils, liquid embolics, and hybrid approaches remain important for complex, recurrent, or anatomically constrained lesions. Emphasis is placed on an anatomy-, flow-, and imaging-based decision framework, cautious interpretation of heterogeneous evidence, and structured long-term surveillance to minimize persistence and optimize clinical outcomes.

## Introduction

Pulmonary arteriovenous malformations (PAVMs) are abnormal direct communications between pulmonary arteries and veins that bypass the capillary bed, resulting in right-to-left intrapulmonary shunting [1]. These shunts allow unfiltered venous blood to enter the systemic circulation, increasing the risk of paradoxical embolization, brain abscess, and hypoxemia [2]. Although many PAVMs are congenital, a substantial proportion are associated with hereditary hemorrhagic telangiectasia (HHT), with PAVMs present in up to 30–50% of affected patients [3]. Population-based thoracic CT screening studies estimate a prevalence of approximately 1 in 2,600 individuals [2].

Endovascular embolization is the standard of care for PAVM treatment, with surgery reserved for select refractory cases [2]. Embolic strategies have evolved from pushable coils to detachable coils, vascular plugs, microvascular plugs, and more recently low-profile braided occluders (LOBOs) [4]. While coil embolization remains effective, recurrence due to recanalization or incomplete nidus exclusion remains a recognized limitation, driving increased adoption of plug-based and distal-feeder-targeted approaches supported by advanced imaging endpoints such as dual-energy CT [4].

Low-profile braided occluders represent an emerging extension of plug technology, enabling deployment within short or tortuous feeders while achieving dense flow stasis, with early studies demonstrating high technical success and complete short-term occlusion [4]. However, current LOBO data remain early and should be interpreted in the context of limited follow-up and potential device-selection bias. This review synthesizes contemporary anatomy-driven embolization strategies and emerging device technologies, with emphasis on a reproducible anatomy-, flow-, and imaging-based framework rather than device preference alone.

## Methods

This narrative review was developed through a literature search of PubMed/MEDLINE and Google Scholar for English-language articles addressing pulmonary arteriovenous malformation embolization, embolic devices, persistence, recurrence, imaging follow-up, and hereditary hemorrhagic telangiectasia. Search terms included combinations of “pulmonary arteriovenous malformation,” “embolization,” “coils,” “vascular plug,” “microvascular plug,” “low-profile braided occluder,” “liquid embolic,” “persistence,” “recanalization,” “nidus embolization,” “venous sac embolization,” “dual-energy CT,” and “surveillance.” Priority was given to systematic reviews, meta-analyses, comparative studies, large retrospective series, device-specific clinical studies, recent imaging studies, and guideline-based publications. Evidence was synthesized narratively according to device class, lesion anatomy, hemodynamics, embolization strategy, imaging endpoints, follow-up considerations, and limitations of the available data.

## Anatomy-, flow-, and imaging-based decision framework

To address the heterogeneity of PAVM anatomy and the limitations of device-to-device comparison, treatment selection can be organized around four practical variables: feeding-artery morphology, nidus-directed or sac accessibility, flow-related migration risk, and the imaging endpoint used to define success. Table 1 explains a summary of the evidence explained in next sections.

**Table 1:** Anatomy-, flow-, and imaging-based framework for pulmonary arteriovenous malformation embolization

Anatomic or hemodynamic setting	Potential strategy	Common devices or adjuncts	Evidence interpretation
Simple PAVM with straight feeder and adequate landing zone	Distal feeding-artery occlusion near the sac	AVP, AVP4, MVP, selected LOBO use	Retrospective data suggest lower persistence with plug-based devices, but anatomy and selection bias limit direct comparison (21–24)
Small, distal, short, or moderately tortuous feeder	Microcatheter-based distal occlusion	MVP, LOBO, detachable coils	Useful when near-nidal deployment is feasible; outcomes may reflect simpler lesions and operator/device selection (4, 22, 24)
Complex, multi-feeder, high-flow, or recurrent PAVM	Nidus/sac-directed or hybrid embolization with flow control	Coils, plug scaffold, n-BCA/Onyx, balloon occlusion, NiFA/VSE	May improve sac or draining-vein regression in selected lesions, but data remain nonrandomized and technique-dependent (25, 31, 32, 35)
Follow-up or suspected persistence	Imaging-based assessment and selective retreatment	TTCE, CTA, dual-energy CT, catheter angiography when intervention is planned	Definitions of persistence and follow-up protocols vary; standardized imaging endpoints are needed (12, 13, 18, 38–42)

*AVP *Amplatzer vascular plug, *AVP4 *Amplatzer vascular plug 4, *CTA *computed tomography angiography, *LOBO *low-profile braided occluder, *MVP *microvascular plug, *n-BCA *n-butyl cyanoacrylate; *NiFA *nidus-plus-feeding-artery embolization, *PAVM *pulmonary arteriovenous malformation, *TTCE *transthoracic contrast echocardiography, *VSE *venous sac embolization

### Anatomy, hemodynamics, and imaging foundations

Pulmonary arteriovenous malformations (PAVMs) are direct communications between one or more pulmonary arterial feeders and a draining pulmonary vein through an aneurysmal sac or nidus. This arrangement bypasses the normal pulmonary capillary bed and creates a right-to-left shunt, explaining the major clinical risks of hypoxemia, paradoxical embolization, and hemoptysis [5]. From a procedural standpoint, the key pre-treatment question is not only whether a PAVM is present, but how the feeder, nidus, draining vein, and surrounding branches are arranged.

### Feeding artery characteristics

Feeding-artery assessment begins with diameter, but diameter alone is insufficient for procedural planning. Tortuosity, angulation, branch points, catheter stability, and the length of usable landing zone determine whether embolization can be performed safely and how close the occlusion point can be placed to the sac [6]. Straight feeders with an adequate landing zone allow more predictable device positioning, whereas short, curved, or branching feeders require more careful distal navigation and deployment planning.

Segmental and subsegmental feeders, particularly in anatomically mobile or angulated regions such as the lingula and right middle lobe, may limit catheter support and make distal positioning more challenging [7]. These anatomic constraints help explain why embolization planning should be individualized rather than based on feeding-artery diameter alone.

### Nidus and sac as the therapeutic target

The nidus or venous sac represents the central shunting component of a PAVM. If embolization is performed too proximally, residual channels, collateral recruitment, or recanalization may allow persistent filling of the sac over time [5, 8, 9]. For this reason, contemporary planning emphasizes understanding the relationship between the feeder, sac, draining vein, and adjacent normal branches before selecting the final embolization point.

### Nidus morphology

**Fig. 1:**
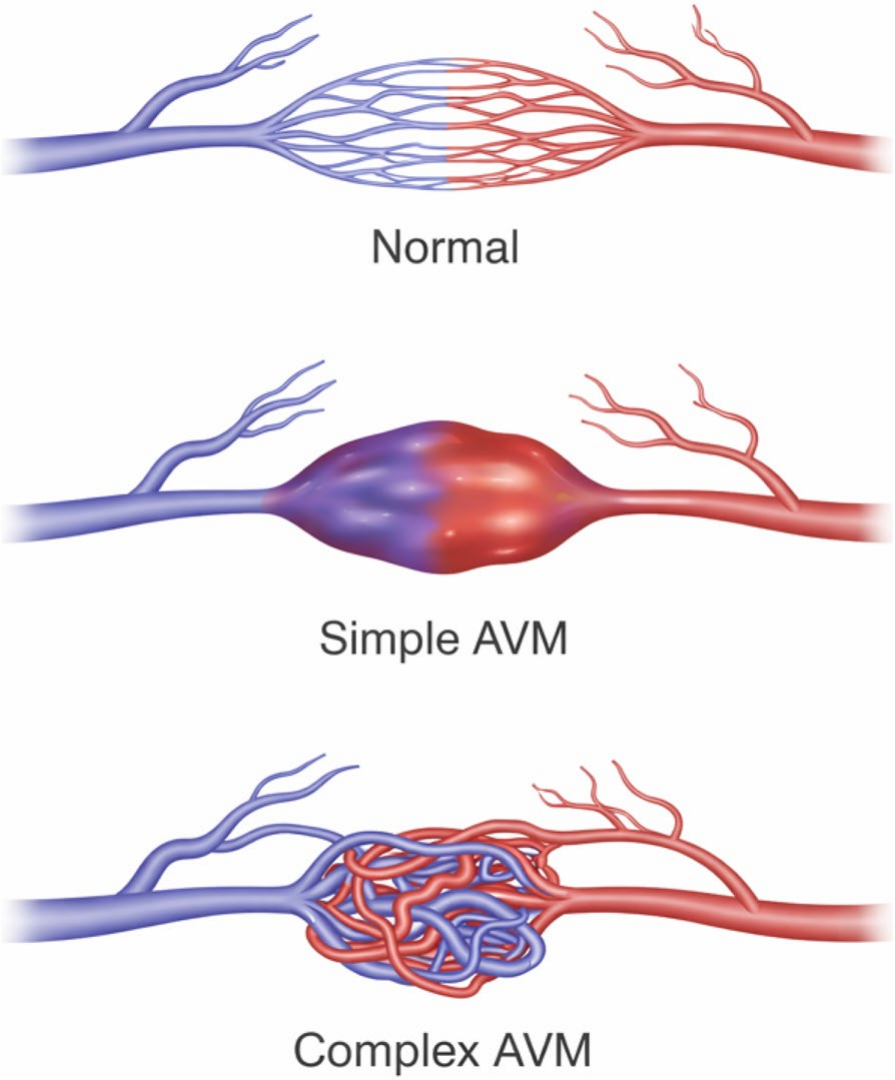
Schematic representation of normal pulmonary vasculature versus simple and complex pulmonary arteriovenous malformations, highlighting direct arterial–venous shunting and increasing nidus complexity

Nidus morphology provides a practical way to describe lesion complexity. Simple PAVMs are generally defined by a single feeding-artery and a single draining vein, whereas complex lesions may have multiple arterial feeders, shared sacs, or veno-venous communications. This distinction is important because complex lesions may require mapping of more than one inflow channel before treatment is considered complete (Fig. 1).

In recurrent or residual PAVMs, inflow patterns can evolve over time, with newly dominant segmental feeders emerging [9]. Preprocedural CT angiography combined with selective pulmonary angiography therefore helps define whether the residual lesion represents recanalization of the treated channel, collateral reperfusion, incomplete distal exclusion, or development of new feeding vessels.

### Hemodynamic considerations and flow control

Hemodynamic assessment complements anatomic assessment. PAVMs may behave as relatively low-flow lesions or as high-flow fistulous communications with rapid transit from pulmonary artery to pulmonary vein. High-flow shunting increases the importance of controlled delivery, stable catheter position, and prevention of embolic migration into the systemic circulation.

Flow-control options include catheter wedging, balloon occlusion, temporary reduction of antegrade flow, scaffold creation, and staged or combined embolization when needed [10, 11]. These techniques are not selected solely by device preference; rather, they are used when flow, vessel caliber, or landing-zone instability makes uncontrolled deployment unsafe.

### Preprocedural imaging and anatomic characterization

Preprocedural imaging defines the working anatomy before embolization. High-resolution CT angiography is used to identify feeding-artery, estimate vessel diameter, evaluate landing-zone length, characterize sac morphology, and assess the draining vein and adjacent normal branches [12]. These findings provide the anatomic map that guides selective angiography and procedural planning.

Follow-up imaging is equally important because technical occlusion at the time of angiography does not always guarantee durable anatomic or hemodynamic exclusion. Persistence may be described by residual sac enhancement, inadequate sac regression, continued right-to-left shunting, or reperfusion through collateral channels, although definitions vary across studies [3, 13]. This variability is one reason standardized surveillance criteria remain important.

### Intraprocedural imaging and device positioning

Intraprocedural imaging can refine the angiographic understanding of complex or overlapping anatomy. Cone-beam CT may help clarify the sac-vein interface, confirm the relationship of the device to the nidus, and identify residual filling that may not be obvious on planar fluoroscopy [14]. When combined with selective angiography, these tools support more precise anatomic localization during embolization [15–17].

Dual-energy CT and other quantitative imaging approaches provide additional ways to evaluate residual perfusion after embolization. Rather than relying only on device appearance or angiographic stasis, these techniques may help distinguish persistent perfusion from expected post-treatment changes and may improve consistency in follow-up assessment [4].

### Advanced imaging endpoints and future surveillance paradigms

Future surveillance paradigms will likely combine anatomic and functional endpoints, including CT angiography, transthoracic contrast echocardiography, dual-energy CT, quantitative perfusion mapping, and standardized sac or draining-vein regression thresholds [4, 18]. The goal is to detect clinically meaningful persistence while limiting unnecessary radiation exposure during lifelong follow-up.

## Embolic devices for PAVM treatment

Multiple embolic devices are currently available for pulmonary arteriovenous malformation management, each offering distinct advantages and limitations based on feeding-artery anatomy, hemodynamic characteristics, and lesion complexity.

### Coils

Coils are metallic embolic devices that occlude vessels by creating mechanical obstruction and promoting thrombosis*.* They remain a foundational embolic agent in PAVM treatment, supported by decades of longitudinal data demonstrating safety and efficacy. Early experience with pushable stainless-steel coils showed that dense packing of the feeding-artery—and, when feasible, the aneurysmal sac—could achieve durable occlusion and meaningful improvements in oxygenation [19]. Subsequent advances, including detachable, fibered, and shape-modified coils, have improved deployment control, packing density, and conformability in short or tortuous feeders.

Despite these refinements, multiple large series and meta-analyses have identified important limitations of coil-only strategies, particularly with respect to long-term persistence. Recanalization may occur through residual flow channels within an incompletely packed coil mass, progressive dilation of the feeding-artery around the coil construct, or collateral recruitment into the sac. Using strict imaging criteria such as < 70% sac regression on CT, persistence rates of approximately 20–40% have been reported in contemporary cohorts [18, 20].

These observations have driven technique optimization, including distal coil deployment near the sac, use of detachable framing coils followed by dense fibered packing, and selective incorporation of adjunctive embolics such as vascular plugs or liquid agents in high-flow lesions. Even in the era of plug-based and braided occluder technologies, coils remain indispensable for very small feeders, sharply angulated anatomy, and as adjuncts to reinforce or extend plug constructs.

### Vascular plugs

Vascular plugs are self-expanding occlusion devices designed to close feeding-arteries with a controlled single-device or limited-device construct. They were developed to overcome limitations of coil embolization by providing a single, self-expanding, low-porosity scaffold capable of reliably occluding high-flow vessels. Among these, Amplatzer Vascular Plugs (AVPs) are widely used in PAVM treatment, particularly for larger-caliber feeding-arteries.

In a single-center comparison of 0.018-inch coils versus plugs for simple PAVMs, Botsford et al. treated 130 lesions with plugs and 182 with coils, achieving 100% technical success in both groups [21]. Despite larger feeders in the plug cohort (4.1 ± 1.6 mm vs 2.8 ± 1.1 mm), plugs demonstrated substantially lower persistence. Using < 30% sac shrinkage, persistence occurred in 7.7% of plug-treated lesions versus 34.1% with coils; using < 70% shrinkage, rates were 16.2% versus 40.1%. Re-embolization was also less frequent with plugs (6.2% vs 28%), with superior 10-year persistence-free survival (81.0% vs 47.3%; HR 0.21; 95% CI 0.09–0.46).

Latif et al. evaluated 393 PAVMs in 112 patients using propensity-weighted analysis comparing coils, AVPs, and microvascular plugs (MVPs) [22]. Technical success was 100% across all devices; however, persistence occurred in 16% of coil-treated lesions, 8% of AVP-treated lesions, and 0% of MVP-treated lesions. Relative to coils, hazard ratios for persistence were 0.37 (95% CI 0.16–0.90) for AVPs and near zero for MVPs, with fewer minor complications observed in plug-treated groups.

More recent experience with AVP4—a low-profile plug designed for feeders ≤ 6 mm—has shown similarly favorable outcomes. In a 10-year series of 51 patients with 103 PAVMs, Cha et al. reported 100% technical success, a 9.7% persistence rate (< 70% sac shrinkage), and a minor adverse event rate of 2.9% [23]. Distal deployment beyond the last normal branch significantly reduced persistence (OR 0.18; 95% CI 0.03–0.81), highlighting the importance of plug position.

### Low-profile braided occluders (LOBO)

Low-profile braided occluders are dense nitinol-mesh occlusion devices that can be delivered through lower-profile systems in selected short or curved feeders. LOBOs represent an evolution of plug-based technology, combining high-density flow occlusion with enhanced conformability in short or curved landing zones. Constructed from braided nitinol mesh, these devices expand to fill the vessel lumen and may achieve rapid, homogeneous stasis in selected anatomy.

Early clinical experience suggests that LOBOs can be delivered through small microcatheters and adapt to noncylindrical vessel segments, including short pedicles near the sac. Yu et al. reported high technical success, dense flow stasis, and complete occlusion on short-term follow-up in PAVM embolization [4]. In a dual-energy CT proof-of-concept study comparing plug-based and coil-only embolization, plug-treated PAVMs—including those treated with LOBOs, AVPs, MVPs, and plug–coil combinations—demonstrated significantly greater reductions in iodine-derived perfusion slopes than coil-only lesions, suggesting more complete hemodynamic occlusion in the studied cohort [4]. However, these findings should be interpreted cautiously because LOBO experience remains limited, follow-up is relatively short, and treatment allocation is influenced by anatomy and operator preference.

The principal potential advantages of LOBOs include dense occlusion with a single device, improved conformability within short or curved feeders, and simplification of embolic constructs in select lesions. Limitations include limited long-term outcome data, higher device cost, and the need for precise sizing and positioning to avoid migration or incomplete flow-channel coverage. Ongoing comparative studies will be essential to define the role of LOBOs relative to AVP4, MVPs, coils, and liquid embolics, particularly in complex or recurrent PAVMs.

### Microvascular plugs (MVP)

Microvascular plugs are detachable covered nitinol devices that provide plug-like occlusion through microcatheters in smaller distal feeding-artery vessels. MVPs extend plug-based embolization into smaller and more distal vessels by enabling plug-like occlusion through microcatheters. These devices consist of a PTFE-covered nitinol framework with a detachable mechanism, allowing precise deployment via 0.027–0.032-inch microcatheters.

In a propensity-weighted analysis, Latif et al. reported 0% persistence with MVPs compared with 16% for coils and 8% for Amplatzer vascular plugs, with similar or fewer minor complications [22]. This finding is encouraging but should not be interpreted as a device-only effect, because MVPs were preferentially used in smaller feeders (mean diameter 2.7 ± 1.1 mm) and more frequently in simple PAVMs. Srinivas et al. compared feeding-artery MVP embolization with combined nidus-plus-feeding-artery coil embolization in 142 PAVMs and demonstrated equivalent technical success (100%) and durable occlusion at both short- and long-term follow-up (≈98% and 89–93%, respectively) [24]. Feeding-artery MVP embolization was significantly less costly (USD 2,599 vs 7,027 per PAVM) and was more commonly used in lesions with smaller feeders (2.3 ± 0.7 mm vs 3.8 ± 1.9 mm). Distal deployment within 1 cm of the nidus was a key predictor of success.

Overall, MVPs appear particularly well suited for small, distal feeders accessible by microcatheter, when near-nidal deployment is feasible and plug-like occlusion is desired without the need for larger delivery systems. Their apparent durability should be interpreted alongside lesion selection, access feasibility, follow-up duration, and institutional technique.

### Liquid embolics (n-BCA/Onyx)

Liquid embolics are injectable agents that polymerize or solidify within the target vessel or nidus to create contiguous occlusion. Primarily, n-butyl cyanoacrylate (n-BCA) and ethylene–vinyl alcohol copolymer (Onyx®), enable deep nidus penetration with contiguous embolization of the feeding-artery, sac, and early draining veins. They are particularly useful in complex PAVMs and in recurrences through dense coil constructs.

Chu et al. reported microballoon-occluded transcatheter n-BCA embolization in 38 PAVMs using a standardized 1:2 n-BCA:Lipiodol mixture and 2.0-F balloon microcatheters, achieving 100% assisted technical success [25]. Complete sac occlusion was achieved in all cases, with 0% persistence on CT at a mean follow-up of 34.5 months and no clinically significant non-target embolization; adjunct coils or AVP4 were required in a minority of cases.

Onyx has been used primarily for coil-recurrent PAVMs. Delpon et al. treated 70 recurrent lesions in 45 patients with HHT using Onyx with or without adjunct coils, achieving 100% technical success and long-term occlusion in approximately 60% at a mean follow-up of 3 years [26]. Recanalization most commonly occurred through pre-existing coil masses, highlighting the technical challenges of retreatment. Acute adverse events were infrequent and generally minor, with no reported major neurologic complications.

Overall, liquid embolics offer the advantage of nidus-level penetration and the ability to augment coil or plug constructs in complex or recurrent lesions. However, their use requires meticulous technique, including effective flow control with catheter wedging or balloon occlusion, careful regulation of injection rate and volume, and heightened awareness of the risk of draining-vein or systemic embolization, particularly in high-flow PAVMs.

Section summary.

Across embolic devices, the available data support a lesion-specific rather than device-exclusive approach. Coils remain supported by long-term experience and are valuable in small or tortuous anatomy, but coil-only constructs have higher reported persistence in several series. Vascular plugs and microvascular plugs may reduce persistence in simple or accessible lesions, although this advantage is partly confounded by anatomy, landing-zone quality, and follow-up definitions. LOBOs and liquid embolics expand options for short, curved, complex, or recurrent lesions, but their long-term comparative evidence remains less mature. Therefore, device choice should be integrated with feeder geometry, nidus accessibility, flow control needs, cost, and surveillance strategy.

## Adjuncts and procedural tools

Endovascular embolization of pulmonary arteriovenous malformations increasingly relies on adjunctive maneuvers that improve catheter stability, enhance flow control, and reduce the risk of persistence.

### Balloon occlusion

Balloon occlusion is a temporary flow-arrest technique used to slow antegrade shunting and improve controlled embolic delivery. It has evolved from a simple anti-reflux maneuver into a key tool for achieving temporary flow arrest during embolization. Early studies demonstrated that balloon catheters reduce non-target embolization and facilitate controlled delivery of liquid agents [27]. The development of dual-lumen and extra-small microballoon catheters has further improved distal navigability and positioning within small or tortuous feeding-arteries [27, 28].

Balloon inflation can reduce antegrade flow by more than 85% within seconds, markedly lowering the risk of embolic migration and venous spillage [27]. This technique is particularly valuable in high-flow PAVMs with feeding-arteries ≥ 3 mm, where stable conditions are essential for safe delivery of liquid embolics such as n-BCA cyanoacrylate [25, 29]. In tortuous anatomy, balloon occlusion also enhances microcatheter stability and minimizes reflux, contributing to shorter procedure times and reduced radiation exposure [8].

In peripheral AVMs, ethanol embolization performed with balloon support achieves 70–100% devascularization in the majority of patients, though complexity increases complication rates [30].

Advanced imaging has further refined balloon-assisted techniques. Cone-beam CT and standardized follow-up CT have revealed residual flow in up to 28% of treated lesions at 6 months, underscoring the need for precise distal embolization [13]. Modern dual-lumen balloons and microvascular plugs now represent the most effective tools for high-flow control and safer distal embolization [22, 27].

### Double microcatheter techniques

Double microcatheter and nidus-directed techniques use one or more microcatheters to treat the nidus/sac and feeder rather than the feeder alone.

Double microcatheter and nidus-plus-feeding-artery (NiFA) techniques were developed to address the higher persistence observed with feeding-artery-only embolization, particularly in complex or large-caliber PAVMs [18, 31]. By enabling combined embolization of the nidus and feeder, NiFA improves occlusion durability when the nidus is accessible or multiple feeders are present.

In a cohort of 219 PAVMs, NiFA achieved higher success than distal feeder embolization (94.3% vs 81.6%; *p* = 0.007) despite greater complexity [31]. Meta-analysis confirms superior efficacy of venous sac embolization ± feeder embolization without increased major complications (OR 3.54; 95% CI 1.66–7.56) [32]. NiFA remains valuable when plug-based deployment is not feasible [24, 33].

### Cone-beam CT (CBCT)

Cone-beam CT has become useful for intraprocedural evaluation of PAVM anatomy because it can clarify the sac–vein interface, confirm distal catheter position, and identify residual filling not apparent on planar angiography. Large series demonstrate technical success > 99%, with treatment success (≥ 70% sac size reduction) ranging from 81–86% depending on device selection [21]. Rather than serving as an independent treatment strategy, CBCT should be viewed as an imaging adjunct that supports near-sac device positioning and standardized outcome assessment [4, 13, 21].

### Anchoring techniques

Anchoring strategies—including scaffold coils, anchor coils, and support catheters—remain important for device stability, particularly in high-flow or complex PAVMs. Their role is most relevant when tortuosity, short landing zones, or high-flow shunting limit the stability of a single plug or coil construct. In these settings, anchoring should be considered a technical adjunct that allows safer distal or hybrid embolization rather than a separate competing treatment paradigm [12, 18, 22, 32, 34].

### Section summary

In summary, adjunctive techniques should be selected according to the technical problem encountered during embolization. Balloon occlusion primarily addresses high-flow migration risk and liquid embolic control; double-microcatheter and NiFA approaches address accessible nidus-directed or sac components; CBCT improves spatial confirmation of distal positioning; and anchoring techniques improve stability when single-device deployment is unreliable. These adjuncts are best viewed as tools that support anatomy- and flow-based embolization rather than independent treatment categories.

## Technique paradigms

Multiple embolization paradigms are used in the treatment of PAVMs, each offering advantages depending on lesion complexity, feeder anatomy, and accessibility of the nidus.

### Feeding-artery-only approach

Feeding-artery-only embolization treats the pulmonary arterial feeder without intentional direct packing of the sac or nidus.

Feeding-artery-only embolization, using coils or plugs, achieves high technical success (≥ 97–100%) across large series [21, 22, 33]. Durability varies across devices and lesion types, with reported persistence generally higher after coil-only embolization (16–47%) than after Amplatzer vascular plugs (0–8%) or microvascular plugs (0–2%) [21, 22, 33]. Because these comparisons are retrospective and influenced by anatomy, feeding-artery-only embolization is best interpreted as a strategy whose success depends on distal deployment, lesion simplicity, and follow-up definition rather than device class alone.

Meta-analyses suggest favorable outcomes with plug-based embolization [18], and ACR guidelines support anatomy-driven device selection [3]. Feeder-only embolization remains appropriate for simple PAVMs when distal occlusion near the nidus is feasible, whereas complex or recurrent lesions may require hybrid or NiFA techniques [20, 24].

### Nidus-focused (NiFA) approach

Nidus-focused and nidus-plus-feeding-artery (NiFA) techniques were developed to address the limitations of feeder-only embolization, particularly in multi-feeder or recurrent PAVMs. Venous sac embolization (VSE) ± feeder embolization is associated with higher odds of achieving ≥ 70% sac or draining-vein reduction (OR 3.54), while NiFA achieves greater success than feeder-only embolization (94.3% vs 81.6%; *p* = 0.007) despite greater complexity [31]. VSE also results in greater vein-size reduction (66.3% vs 46.4%; *p* = 0.009) [35]. NiFA techniques are therefore best suited for complex lesions, larger feeders, and cases with accessible nidus anatomy.

### Hybrid (feeder + nidus) approach

Hybrid approaches combine feeder and nidus-directed or venous sac embolization to address complex inflow patterns. Meta-analysis of 298 lesions showed venous sac embolization (VSE) ± feeder embolization was superior to feeder-only embolization for achieving ≥ 70% sac or draining-vein reduction (OR 3.54; 95% CI 1.66–7.56) without increased major complications [32]. Nagai et al. reported 0% reperfusion with VSE versus 50% after feeder-only treatment [35]. These findings support hybrid techniques for multi-feeder, high-flow, unstable, or recurrent PAVMs, although the evidence remains nonrandomized and operator-dependent [18, 31].

### Section summary

Taken together, the major technique paradigms differ less by device name than by the anatomic target of occlusion. Feeder-only embolization is appropriate for simple lesions when durable distal occlusion is achievable, whereas nidus-focused, venous sac, or hybrid approaches are most relevant for complex, high-flow, recurrent, or multi-feeder lesions. The current evidence favors distal and lesion-targeted embolization, but comparisons remain limited by retrospective design, variable persistence definitions, and operator-dependent execution.

## Device selection and comparative outcomes

Device selection in PAVM embolization is guided by anatomic, hemodynamic, and procedural factors, with the goal of achieving durable occlusion while minimizing complications and reintervention [22].

In simple PAVMs with straight feeders and adequate landing zones, available retrospective data suggest lower persistence and re-embolization after vascular plug-based embolization than after coil-only strategies, even when plug-treated feeders are larger [21].

Microvascular plugs may extend plug-like occlusion to smaller or moderately tortuous feeders, but their outcomes are influenced by lesion selection and feasibility of near-nidal deployment [22, 24].

**Fig. 2:**
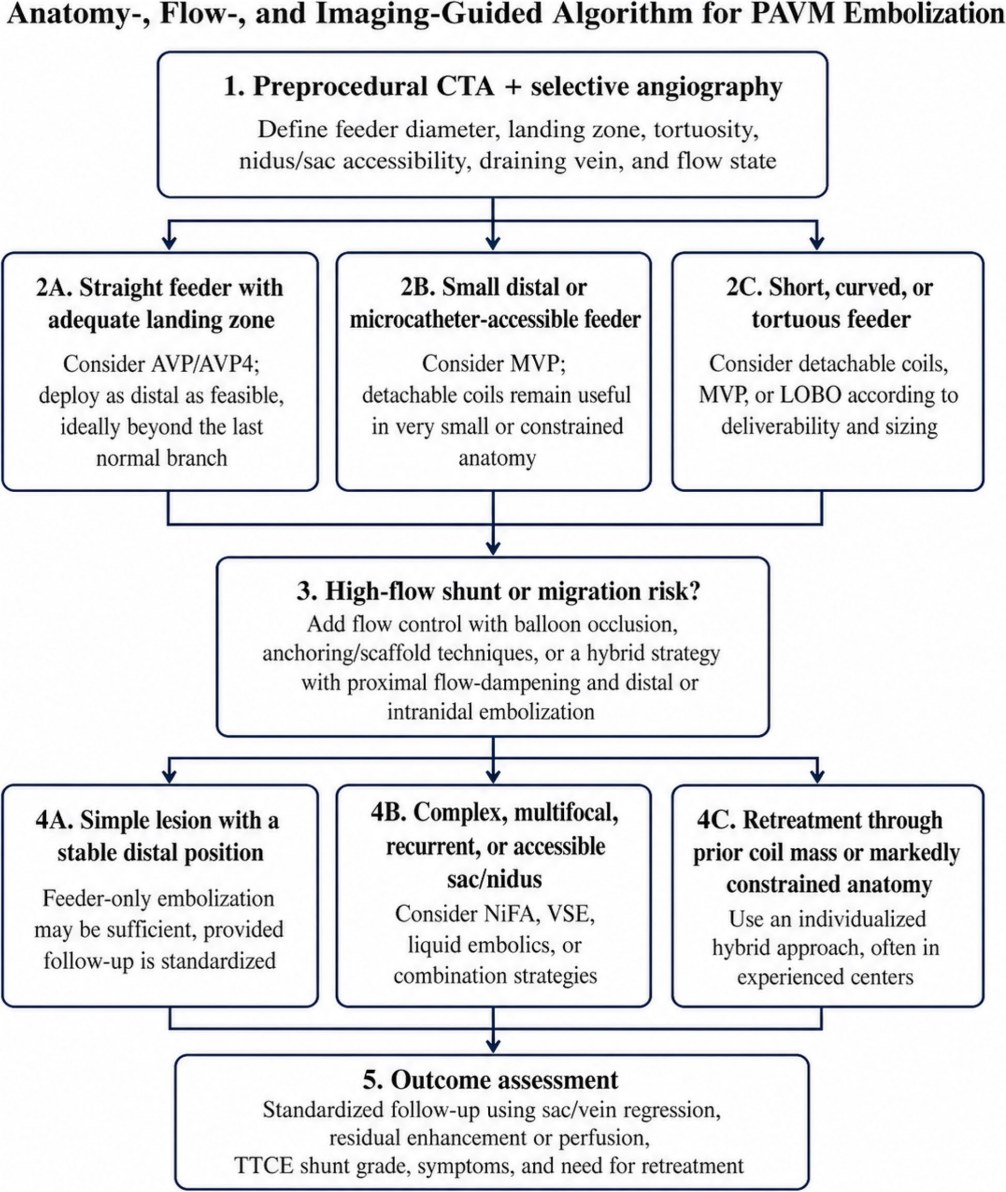
Proposed anatomy-, flow-, and imaging-based decision algorithm for pulmonary arteriovenous malformation embolization. The algorithm emphasizes lesion-specific strategy selection and cautious interpretation of outcomes in the context of retrospective evidence, follow-up definitions, and operator-dependent technique

Coils remain important in anatomically constrained settings, including short, small, or highly tortuous feeders, while liquid embolics and hybrid strategies are generally reserved for complex, recurrent, or high-flow lesions requiring advanced flow control [5, 8, 25, 36]. Overall, the evidence supports an anatomy- and flow-based device-selection paradigm rather than a universal hierarchy of embolic agents (Fig. 2).

## Clinical outcomes and follow-up

Endovascular embolization of PAVMs achieves high technical success (97–100%) across devices and techniques (21, 22), but long-term durability varies by embolic choice, anatomy, technical endpoint, and follow-up definition. Coil-based embolization has been associated with higher persistence (16– > 40%), whereas vascular and microvascular plug series often report lower persistence, frequently < 10%, with favorable persistence-free survival [21, 22]. These data should be interpreted cautiously because device choice is rarely randomized and may reflect lesion simplicity, feeder size, landing-zone quality, and operator preference. Reintervention most commonly results from collateral reperfusion or recanalization and may be reduced by distal, nidus-directed embolization compared with proximal feeder-only approaches [31, 36]. Repeat embolization remains effective when residual anatomy is well defined. Overall, modern strategies yield favorable long-term outcomes with low complication rates when anatomy and follow-up findings guide retreatment [3].

## Complications and mitigations

PAVM embolization is generally safe but carries recognized risks, including paradoxical air or thromboembolic stroke or TIA, device migration into the pulmonary veins or systemic circulation—particularly in high-flow or undersized deployments—reperfusion or recanalization, pulmonary infarction or pleuritic chest pain after distal embolization, access-site complications, and contrast-related nephropathy or allergy [8, 15, 37].

Risk mitigation strategies include meticulous air management, appropriate device sizing with plug-based oversizing (typically 20–50%), and distal device deployment near the sac or beyond the last normal branch to reduce persistence [20, 23]. Balloon occlusion and liquid embolics should be reserved for experienced centers due to non-target embolization risk. With careful technique and post-procedural monitoring, major complication rates remain low, and most adverse events are minor and self-limited.

## Surveillance and long-term management

Recent studies emphasize the need for lifelong surveillance after PAVM embolization, particularly in patients with hereditary hemorrhagic telangiectasia (HHT), who remain at risk for recanalization, reperfusion, and development of new lesions [3]. Surveillance imaging is typically recommended at 6–12 months post-embolization and every 3–5 years thereafter, as late reperfusion may occur years after treatment and predispose to stroke, brain abscess, or progressive hypoxemia [12, 20, 22, 35].

Transthoracic contrast echocardiography (TTCE) is increasingly favored for screening and follow-up because of its near-100% sensitivity for right-to-left shunts and absence of radiation exposure [38]. In adults with HHT, patients without baseline shunts may be re-screened at longer intervals, whereas those with detectable shunts require closer surveillance every 3–5 years [39]. In children, TTCE-based surveillance is recommended during growth and after embolization due to higher rates of reperfusion [40, 41]. TTCE is also preferred during pregnancy for fetal safety [40].

CT angiography (CTA) remains the gold standard for anatomic assessment, documenting sac regression, recanalization, or new feeders using standardized metrics such as ≥ 70% sac or vein reduction [12]. CTA is generally performed at 6–12 months and periodically thereafter when TTCE shows moderate or large shunts, symptoms recur, or neurologic events occur [12, 32, 42]. Radiation exposure considerations are particularly important in pediatric and pregnant patients, favoring TTCE-first strategies and low-dose CTA when necessary [3, 42].

Catheter pulmonary angiography is now reserved primarily for planned intervention, serving as the definitive anatomic reference at the time of embolization [12].

## Limitations, controversies, and evidence gaps

Several limitations should temper interpretation of the available evidence. Most comparative data are retrospective, single-center, and influenced by device-selection bias, because plugs and microvascular plugs are often used in anatomically favorable lesions, while coils, liquid embolics, or hybrid approaches are frequently used in tortuous, recurrent, or complex PAVMs. Randomized controlled trials directly comparing contemporary embolic devices or techniques are lacking. Definitions of persistence also vary substantially across studies, ranging from residual enhancement to different sac-regression thresholds, including < 30% versus < 70% reduction. Follow-up protocols differ in imaging modality, timing, radiation exposure, and duration, limiting cross-study comparisons. Operator experience, institutional preference, device availability, and cost may also influence both technique selection and outcomes. Patient-centered outcomes, including symptom burden, quality of life, reintervention burden, and cumulative radiation exposure, remain underreported and should be incorporated into future comparative studies.

## Future directions and conclusions

Endovascular embolization is the standard treatment for PAVMs, with durability driven by anatomy, flow, imaging endpoints, and device selection. Contemporary evidence supports a lesion-specific strategy: plug-based and microvascular plug approaches appear durable in anatomically suitable lesions, while coils, liquid embolics, and hybrid or nidus-directed approaches remain important for complex, recurrent, or anatomically constrained PAVMs. However, the evidence base remains limited by retrospective design, selection bias, variable definitions of persistence, heterogeneous imaging follow-up, and operator-dependent technique. Future work should focus on standardized imaging endpoints, prospective comparative studies, cost-effectiveness analyses, patient-centered outcomes, and structured lifelong surveillance to minimize recurrence and sustain clinical benefit.

## Data Availability

Not applicable. No new datasets were generated or analyzed for this review. All data discussed are derived from previously published studies cited in the manuscript.
